# Inflammatory Mechanisms in Myocarditis—Recent Therapeutic Strategies

**DOI:** 10.3390/biom15101475

**Published:** 2025-10-20

**Authors:** Stergios Soulaidopoulos, Dimitris Tousoulis, Marios Sagris, Svetlana Aghayan, Konstantinos Platanias, Alexios Giannakodimos, Emilia Lazarou, Konstantinos Tsioufis, George Lazaros

**Affiliations:** First Cardiology Department, Hippokration Hospital, Athens Medical School, National and Kapodistrian University of Athens, 11527 Athens, Greece; soulaidopoulos@hotmail.com (S.S.); masagris1919@gmail.com (M.S.); dr.svetlana.aghayan@gmail.com (S.A.); konstantinosplatanias99@gmail.com (K.P.); alexisgiannak@hotmail.com (A.G.); emilazarou@gmail.com (E.L.); ktsioufis@gmail.com (K.T.); glaz35@hotmail.com (G.L.)

**Keywords:** myocarditis, inflammation, cardiomyopathy, immunosuppression, immunomodulation

## Abstract

Myocarditis is an inflammatory disease of the heart characterized by a complex interplay between innate and adaptive immune responses. The innate immune system provides first-line defense and includes soluble molecules, including macrophages, neutrophils, dendritic cells, and molecular mediators, but lacks immunological memory. In contrast, the adaptive immune system, via T and B lymphocytes, offers high specificity and long-term memory, which can sometimes target myocardial tissue, causing autoimmune injury. Particularly, acute myocarditis is characterized by dysregulated immune signaling, with cytokines (IL-2, IFN-γ, IL-12, IL-4, IL-10) and chemokines (MCP-1, CXCL4, CXCL10) driving disease progression, while adhesion molecules (ICAM-1, VCAM-1, VAP-1) promote leukocyte trafficking and cardiac inflammation. The balance between pro-inflammatory and regulatory responses determines disease outcomes, ranging from resolution with recovery to fulminant myocarditis or progression to dilated cardiomyopathy. Emerging therapeutic approaches targeting cytokines, chemokines, and adhesion molecules, along with established immunosuppressive treatments, underline the potential for modulating immune responses in myocarditis and, thereby, improving patient outcomes.

## 1. Introduction

Myocarditis represents an inflammatory disease of the myocardium, which can be triggered by a plethora of infectious and non-infectious causes. The diagnosis of myocarditis may be often quite challenging, owing to its presentation with a wide spectrum of possible clinical scenarios [1]. For many years, endomyocardial biopsy (EMB) was considered the standard method for diagnosing and classifying myocarditis, based on a combination of established histological, immunological, and immunohistochemical criteria [2,3]. The introduction of advanced imaging techniques, namely cardiac magnetic resonance (CMR), in daily clinical practice, has however, changed the way myocarditis is diagnosed in the majority of cases. Particularly, the detection of cardiac inflammation on CMR along with elevated troponin levels indicating myocardial injury in the presence of relevant clinical symptoms and signs, is usually sufficient for the non-invasive confirmation of the diagnosis in the majority of cases [4,5].

Although determining the etiology may not be necessary in cases with mild clinical presentation, precise identification of the underlying cause through an understanding of the activated pathogenetic pathways may be crucial for effective management and improved patient outcomes in cases with more critical clinical presentation. Indeed, different etiologies trigger distinct immune responses involving various cellular and molecular mechanisms [6]. In addition to this, certain inflammatory markers correlate with disease severity and prognosis, enabling improved risk stratification and treatment monitoring [7]. Overall, a deeper understanding of these mechanisms facilitates the development of targeted therapies and helps prevent long-term complications of myocarditis, such as myocardial fibrosis, arrhythmias, and heart failure [8,9].

Within this context, the scope of this article is to review the inflammatory pathogenetic mechanisms involved in myocarditis and to summarize current therapeutic strategies.

## 2. Epidemiology, Etiopathogenesis, and Diagnosis

Given the infrequent use of EMB for diagnostic confirmation, it is difficult to determine the actual incidence of myocarditis. In addition to this, the diagnosis is frequently missed owing to subtle clinical manifestations. It is estimated that the global incidence of acute or subacute myocarditis is about 500,000 cases per year, being responsible for about 30,000 deaths [10]. Among patients diagnosed with heart failure, the incidence of myocarditis varies between 0.5% and 4%, while it is estimated at about 3% among patients presenting with chest pain at the emergency department [11,12].

A plethora of infectious and non-infectious agents may cause acute myocarditis. Viral infections represent the most common cause, with classic enteroviruses, adenoviruses, parvovirus B19, and human herpesvirus 6 being more frequently detected when EMB is performed [7,13]. However, non-viral pathogens, such as bacteria and parasites, may also cause myocardial injury either directly or indirectly by the activation of catastrophic autoimmune responses [9].

Multiple pharmacological agents, such as antipsychotics immunotherapies, and vaccines, may display toxic effects on the myocardium, which may be characterized by a diversity of the underlying pathogenetic mechanisms [14]. For example, antipsychotic drugs are commonly associated with hypersensitive reactions that manifest as eosinophilic myocarditis [15]. Myocarditis related to immunotherapies, on the other hand, is driven by T cell-mediated immune checkpoint inhibition, presenting as fulminant lymphocytic myocarditis [16]. Finally, vaccine-mediated myocarditis, which has recently gained increasing interest in the context of COVID-19, seems to be associated with strong innate immune responses secondary to vaccine mimicry of myocardium antigens [17,18].

Regardless of the triggering cause, the activation of autoimmunity, characterized by the presence of heart-reactive autoantibodies, is a prominent feature of all forms of acute or chronic myocardial inflammation [19]. In these terms, myocarditis demonstrates a notable association with systemic autoimmune diseases. The incidence of myocarditis, for instance, may reach 18% among patients with systemic lupus erythematosus [20]. Sarcoidosis, which is characterized by the formation of non-necrotizing granulomas in various organs, seems to manifest in 5% of patients as clinically overt inflammatory infiltration of the myocardium, while in approximately 25% of asymptomatic patients, cardiac involvement is verified by autopsy or imaging studies [21]. Giant cell myocarditis is characterized by T-cell-mediated inflammation of the myocardium and is also considered to be of autoimmune origin, given its association with disorders such as Hashimoto thyroiditis, myasthenia gravis, rheumatoid arthritis, and inflammatory bowel disease [22].

The development of viral or autoimmune myocarditis and its progression to dilated cardiomyopathy are likely influenced by genetic factors. Indeed, not all patients exposed to infectious agents or affected by autoimmune diseases develop myocarditis, and only a small proportion ultimately progress to dilated cardiomyopathy. This progression appears to occur predominantly in cases characterized by uncontrolled, persistent chronic inflammation and the presence of pathogenic cardiac autoantibodies targeting myocardial structural, sarcoplasmic, or sarcolemmal proteins.

The recently published ESC guidelines have introduced the term “inflammatory myopericardial syndrome”, encompassing a broad spectrum of inflammatory conditions affecting both the myocardium and pericardium [23]. It is recommended to use this term as an umbrella diagnosis during the initial workup, guided by the recognition of diagnostic red flags, including clinical, serological, and imaging biomarkers. The identification of these features should raise clinical suspicion and subsequently be followed by a risk stratification based on the clinical presentation and the assessment of LV function by cardiac imaging. According to the proposed classification, stable and oligosymptomatic, low-risk patients may be safely discharged. In contrast, patients with heart failure symptoms, arrhythmias, or impaired left ventricular function are considered either intermediate or high risk and should be hospitalized for appropriate management, including heart failure therapy or biopsy-guided treatment. Particularly, EMB should be performed in patients with high-risk clinical features, such as advanced heart failure symptoms, arrhythmias, and significant LV dysfunction, to enable precise identification of the histological subtype of cardiac inflammation and allow targeted therapy.

## 3. Inflammatory Mechanisms

### 3.1. Innate and Adaptive Immunity Regulation

The innate immune system constitutes the first line of defense against pathogens. It includes physical barriers such as the skin, as well as soluble and cellular components including cytokines, chemokines, complement proteins, macrophages, neutrophils, and dendritic cells. Although rapid and effective, this system does not generate immunological memory. In contrast, the adaptive immune system is characterized by high specificity and the capacity to develop immunological memory, enabling faster and more robust responses upon re-exposure to previously encountered pathogens. The principal regulators of this response are T and B lymphocytes [23].

In acute myocarditis, an imbalance in the coordination between innate and adaptive immune mechanisms has been reported [24]. Following recognition of pathogens by pattern recognition receptors (PRRs)—notably Toll-like receptors (TLRs) and NOD-like receptors (NLRs) [25]—the innate immune system releases cytokines, chemokines, and chemoattractants that recruit mast cells, neutrophils, dendritic cells, monocytes, and macrophages to the injured myocardium [23,24]. Among these, macrophages play a pivotal role through phagocytosis, cytotoxicity, and cytokine release. A key mediator is the NLRP3 inflammasome, a multiprotein complex composed of NLRP3 (NOD-like receptor family, pyrin domain–containing 3), ASC (apoptosis-associated speck-like protein containing a caspase recruitment domain), and pro-caspase-1 [26]. Upon activation by pathogen-associated molecular patterns (PAMPs) or danger-associated molecular patterns (DAMPs)—particularly after myocardial injury—the inflammasome triggers caspase-1 activation and subsequent release of pro-inflammatory cytokines IL-1β and IL-18, amplifying the inflammatory response [26,27].

The adaptive immune response provides a broad repertoire of antigen receptors capable of recognizing diverse pathogens. However, dysregulation may result in misidentification of cardiac antigens as foreign, leading to autoimmunity. While immune activation is generally protective, excessive or unresolved inflammation can promote cardiomyocyte injury, ventricular remodeling, and progressive dysfunction [28,29]. Following myocardial injury, intracellular antigens released from necrotic myocytes may be inadequately cleared and are instead processed by antigen-presenting cells (APCs) [30]. These antigens, when presented via major histocompatibility complex (MHC) molecules, activate antigen-specific T and B lymphocytes, driving their differentiation into cytotoxic T lymphocytes (CTLs), helper T cells (Th), plasma cells, and memory cells. These subsets mediate complementary immune functions: CTLs exert cytotoxic activity, Th cells coordinate cytokine signaling, plasma cells secrete antibodies, and memory cells sustain long-term immunological surveillance [30].

During the acute phase of myocarditis, dysregulated adaptive responses may produce self-reactive T and B lymphocytes that specifically target myocardial tissue. In most cases, regulatory T lymphocytes (Tregs) restore immune homeostasis, leading to gradual recovery of left ventricular (LV) contractility, resolution of arrhythmias, and reduction in inflammatory markers [9]. However, when this regulation is insufficient, persistent inflammation may result in fulminant myocarditis, characterized by cytokine storm and cardiogenic shock, or in chronic inflammation progressing to dilated cardiomyopathy (DCM).

### 3.2. Molecular Pathways

The molecular mechanisms underlying myocarditis involve adhesion molecules, cytokines, and chemokines, which collectively regulate immune cell activation, recruitment, and tissue injury.

Cytokines play a central role in orchestrating the immune response during myocarditis. They are broadly classified into Th1 cytokines, including IL-2, IFN-γ, IL-12, and TNF-α, and Th2 cytokines, such as IL-4, IL-5, IL-6, and IL-10 [23]. Using myosin-induced myocarditis models in rats, Ayach et al. demonstrated that the acute phase is characterized by a dominant Th1 response, with elevated IL-2 and IFN-γ, whereas during the convalescent phase, Th2 cytokines such as IL-10 predominate [3]. Similarly, Izumi et al. confirmed that IL-2 and IL-12 are pivotal regulators across both acute and convalescent phases of the disease [24].

IFN-γ appears to act protectively, as demonstrated by Eriksson et al., who found that impaired IFN-γ receptor signaling coupled with elevated IL-12 aggravated myocarditis and led to increased mortality [5]. Barin et al. further reported that fatal eosinophilic myocarditis developed in the absence of both IFN-γ and IL-17A [25]. In contrast, IL-12 has been associated with the differentiation of pathogenic CD8^+^ T-cell effectors, which promote myocardial injury [26]. Consistent with this, mice lacking the IL-12 receptor or the IL-12p40 ligand exhibited markedly reduced IFN-γ expression and were protected from myocarditis [27].

The role of IL-4 and IL-10 remains more complex. Both cytokines promote B-cell antibody production, yet studies report conflicting outcomes in myocarditis. Roffe et al. showed that IL-10 reduced parasite burden and protected against fatal myocarditis in a *Trypanosoma cruzi* mouse model [28], whereas Cen et al. found that IL-10–producing B cells were increased in Coxsackievirus B3-induced acute viral myocarditis [29]. Similarly, Afanasyeva et al. demonstrated that neutralization of IL-4 protected against myocarditis and enhanced IFN-γ expression [30], while other studies indicated that sustained IL-4 and IL-17 elevation predicted progression from viral myocarditis to dilated cardiomyopathy (DCM). Conversely, Li et al. reported that IL-4 suppressed matrix metalloproteinase activity and improved cardiac function in murine myocarditis, highlighting its potential cardioprotective role [31].

Chemokines are another essential component of the immune response, functioning in chemotaxis, leukocyte degranulation, hematopoiesis, and angiogenesis. They are structurally categorized into CXC, CC, XC, and CX3C families based on the positioning of conserved cysteine residues and act through binding to G protein–coupled receptors, which makes them attractive therapeutic targets [32]. In experimental viral myocarditis, Coxsackievirus B3 infection was shown to modulate chemokine expression in cardiomyocytes, with monocyte chemoattractant protein-1 (MCP-1) playing a critical role during the early stages [33]. Furthermore, the natural compound myricetin was shown to suppress autoimmune responses and reduce MCP-1 expression in cardiomyocytes, indicating its potential as a therapeutic agent [34]. Additional pathways have also been implicated. Asaumi, Y. et al. identified the CXCL13/CXCR5 axis as a contributor to myocarditis pathogenesis [35], while Krueger, G.R. et al. revealed that the CXCL4/CXCR3 axis regulates cardiac fibrosis via TGF-β1/Smad2/3 signaling in viral myocarditis [36]. Furthermore, CXCL10 was shown to inhibit viral replication by recruiting natural killer cells in Coxsackievirus B3-induced myocarditis [37]. More recently, Manaresi et al) demonstrated that in immune checkpoint inhibitor (ICI)-related myocarditis, the CXCR3–CXCL9/10 axis plays a critical role in driving myocardial inflammation, and inhibition of this pathway reduced inflammation and improved survival [38].

Adhesion molecules also contribute significantly to disease pathogenesis by regulating leukocyte trafficking, cell–cell interactions, and cell–matrix adhesion. The principal families involved include integrins, selectins, and members of the immunoglobulin superfamily [39]. Intercellular adhesion molecule-1 (ICAM-1), a ligand for lymphocyte function–associated antigen-1 (LFA-1), is markedly upregulated in murine models of acute Coxsackievirus B3-induced myocarditis and remains elevated even during immunosuppressive therapy, suggesting a role in chronic myocardial inflammation. Similarly, vascular cell adhesion molecule-1 (VCAM-1) expression is increased in autoimmune myocarditis, though not directly correlated with disease severity. More recently, vascular adhesion protein-1 (VAP-1), a membrane-bound molecule mediating leukocyte adhesion and transmigration, has been studied as a novel imaging biomarker, with VAP-1–targeted PET successfully identifying myocardial lesions in autoimmune myocarditis [40]. Table 1 summarizes the main pathogenetic pathways involved in acute myocarditis.

## 4. Therapeutic Strategies for Myocarditis

### 4.1. General Treatment Measures

Although the therapeutic strategy for myocarditis is directed by the underlying cause of the disease and often requires multispecialty input, general measurements are applied based on the presence of complications and especially the hemodynamic instability of the patient, the reduction in left ventricular ejection fraction, and the appearance of arrhythmias. Acute forms of myocarditis often have uncertain progress, and hospital admission is generally required for clinical observation of the patient, diagnosis investigation, and surveillance of complications [24,25]. Relaxation, monitoring of the vital signs, execution of electrocardiograms, and often pain management with conventional therapies are the basic approach in an uncomplicated form of acute myocarditis with preserved ejection fraction. Conventional pain management includes paracetamol, while use of nonsteroidal anti-inflammatory drugs (NSAIDs) should be administered in cases of pericardium involvement or persistent chest pain, as they are now considered safe for use in this setting [26]. Other anti-inflammatory drugs such as colchicine are recommended in patients with myopericarditis [27]. Physical activity as well as exercise testing are contraindicated during the acute phase of myocarditis, while the duration of exercise restriction ranges between 3 and 6 months [28]. General use of immunosuppressive drugs failed to prove clinical benefits and is restricted to certain indications, such as severe patients with giant cell or non-viral myocarditis. However, when patients present with complications, especially left ventricular systolic dysfunction, cardiac arrhythmias, and hemodynamic instability, admission to intensive care units is considered necessary.

The initial treatment of hemodynamically stable patients with reduced LVEF is based on heart failure (HF) guidelines. Early initiation of HF drugs with proven survival benefits, such as beta-blockers, angiotensin-converting enzyme inhibitors or angiotensin receptor–neprilysin inhibitors, aldosterone antagonists, and sodium–glucose cotransporter 2 inhibitors, is considered crucial and suggests potential benefits [29,30]. Appropriate use of diuretics is also recommended when symptoms and signs of HF congestion are present. No specific recommendations exist regarding transient reduction in LVEF; however, maintenance of HF treatment seems to be beneficial when CMR preserves myocardial Late Gadolinium Enhancement (LGE) [24].

Management of cardiac arrhythmias is considered of greatest importance in the treatment of myocarditis. Myocarditis caused by sarcoidosis, giant cells, or Lyme disease typically leads to both atrioventricular blocks and tachyarrhythmias. Supportive measures are usually applied while conduction disturbances and ventricular arrhythmias tend to be reversible after the acute phase of myocarditis [31]. No specific recommendations are established; close monitoring of the patients, beta-blockers, and antiarrhythmic agents typically consists of the treatment for premature ventricular contractions, supraventricular and ventricular arrhythmias [32]. Implantation of implantable cardioverter-defibrillators (ICDs) is still debatable. ICDs are generally not recommended while the arrhythmogenic risk decreases after the acute phase of the disease. Alternative strategies using wearable defibrillator vests could be applied for the prevention of malignant arrhythmias during the early stage of myocarditis [33]. Secondary prevention of sudden cardiac death using ICDs should be considered in specific cases of the disease, taking into consideration the history of the patients, imaging results, and the type of myocarditis. It is worth mentioning that in the ITAMY registry, the percentage of ICD implantation in patients after acute myocarditis and preserved left ventricular systolic function was only 1.6% [34].

Cardiogenic shock and hemodynamic instability are considered crucial complications of myocarditis, rendering necessary the immediate transport of the patients to intensive care units for administration of inotropic agents and access to mechanical circulatory support systems. Ventricular assist devices (VADs) and extracorporeal membrane oxygenation (ECMO) systems alone or in combination with devices reducing left ventricular afterload and intracavitary pressures could support hemodynamic circulation, offering myocardial recovery, ameliorating biventricular unloading and coronary perfusion, and providing a successful bridge to permanent recovery or long-term left ventricular assist devices and heart transplantation [5]. Inability to wean off the mechanical circulatory support systems signals the possibility for long-term VAD placement and heart transplantation [35].

### 4.2. Antiviral Treatment for Myocarditis

To date, randomized clinical trials have not confirmed the therapeutic effectiveness of antiviral treatments for all causes of acute viral myocarditis [9]. Regarding patients with latent infections caused by HHV-6 or parvovirus B19 (B19V), no specific antiviral therapy has been established. In cases of acute myocarditis associated with active HHV-6, B19V, adenovirus, or enterovirus infections, antiviral agents such as ganciclovir or valganciclovir have been proposed; however, their clinical efficacy remains uncertain, and evidence is limited to small, heterogeneous studies [23]. Consequently, antiviral treatment in myocarditis remains controversial and should be considered on an individual basis. Interferon beta immunoglobulins may also prove useful [24]. In patients with latent myocarditis caused by Epstein–Barr virus or cytomegalovirus, antiviral therapy targeting HSV may also help lower viral load [24]. Although the effectiveness of aciclovir, ganciclovir, or valaciclovir in myocarditis patients has not been directly assessed, their use may generally be considered in HSV myocardial infections [36]. In cases of enteroviral myocarditis in neonates, treatment with antiviral agents such as pocapavir and pleconaril, along with intravenous immunoglobulin (IVIG) therapy, has shown effectiveness [37]. Concerning B19V, IVIG is commonly administered to patients with severe viremia and associated clinical complications. Emerging antiviral approaches for B19V infection, currently under investigation, include synthetic nucleotide analogs such as cidofovir and brincidofovir, flavonoids, and hydroxyurea [38]. Nevertheless, no established treatments currently exist for B19V-associated inflammatory cardiomyopathy, and it is generally suggested that treatment is not required when low levels of B19V DNA are found in cardiac tissue without evidence of myocardial inflammation [28]. Findings from small observational studies suggest that immunosuppressive therapy may prove useful for patients with low myocardial B19V DNA levels and ongoing cardiac inflammation, as shown in the CaPACITY program. Additionally, patients with B19V RNA positivity have shown improvement following treatment with the antiviral agent telbivudine, likely due to its immunomodulatory effects [39].

Established antiviral therapies are used to manage myocarditis or inflammatory cardiomyopathy linked to HIV, HCV, or influenza infections. These include antiretroviral regimens for HIV-associated myocarditis, as well as a combination of ombitasvir, paritaprevir, ritonavir, and dasabuvir for HCV-related cases; neuraminidase inhibitors such as peramivir and zanamivir may also be administered for influenza-associated myocarditis [40,41,42]. Moreover, in regard to COVID-19-related myocarditis, numerous antiviral treatment strategies are currently being explored. These include agents that block viral entry into host cells, such as chloroquine, hydroxychloroquine, camostat mesylate, and umifenovir; protease inhibitors, such as lopinavir–ritonavir and darunavir; RNA polymerase inhibitors, like remdesivir; as well as anti-cytokine therapies targeting IL-6 and IL-1β pathways [43].

Experimental studies in animals have demonstrated that both IFN-α and IFN-β reduce viral replication and myocardial injury, with IFN-β proving more effective than IFN-α in fully eradicating the cardiac viral load [44]. Both spontaneous and IFN-β–induced viral clearance have been linked to clinical and hemodynamic improvement in myocarditis patients, while ongoing viral presence is associated with adverse outcomes due to progressive left ventricular dysfunction [45]. Notably, the phase II BICC clinical trial examined the impact of IFN-β therapy on viral clearance in patients with inflammatory cardiomyopathy and persistent myocardial viral infection caused by adenovirus, enterovirus, or B19V; patients with myocarditis positive for enterovirus or adenovirus, confirmed via endomyocardial biopsy, experienced viral clearance following IFN-β treatment. However, this therapy did not result in viral DNA clearance in individuals with B19V-positive myocarditis [46]. Further research is needed to determine whether combining antiviral and immunosuppressive therapies could be beneficial for selected patients with virus-positive myocarditis depending on the stage of the disease.

## 5. Immunosuppressive and Immunomodulatory Therapy

The role of immunosuppressive and immunomodulatory therapy in myocarditis remains controversial. In the absence of a clearly defined viral etiology, particularly when active viral replication has been excluded, such therapies may be considered in selected cases. Their use is most justified in myocarditis of confirmed autoimmune origin, as established through myocardial biopsy, serological testing, and PCR analysis. Immunosuppressive treatment may also be appropriate in the presence of histological evidence of active myocardial inflammation or in specific subtypes of myocarditis, such as giant cell, sarcoid, eosinophilic, or immune checkpoint inhibitor (ICI)–induced myocarditis. However, the efficacy of these approaches in viral or idiopathic forms remains uncertain, and thus, treatment decisions should be individualized based on the underlying pathophysiology and clinical presentation [23].

### 5.1. Glucocorticoids

Glucocorticoids are the first-line agents in primary autoimmune myocarditis, particularly in giant cell and sarcoid variants. The recommended initial dose is 1 mg/kg/day. Randomized controlled trials have demonstrated improvements in LVEF in patients with virus-negative myocarditis treated with Prednisolone in combination with Azathioprine [47].

### 5.2. Azathioprine

Azathioprine is a purine nucleoside analog that inhibits lymphocyte proliferation. It is predominantly used in combination with glucocorticoids. Clinical studies have shown that this combination results in improved LVEF and a reduction in heart failure symptoms [48,49].

### 5.3. Cyclosporine A/Tacrolimus

These agents act by inhibiting T-cell activity and are particularly effective in giant cell myocarditis. Case reports have documented clinical improvement and symptom reduction when Cyclosporine is administered in combination with glucocorticoids, especially with giant cell myocarditis [50,51].

### 5.4. Mycophenolate Mofetil (MMF)

MMF has seen increasing use in recent years and is generally well tolerated. It is employed in cases of sarcoid myocarditis. In patients with autoimmune, virus-negative myocarditis, substitution of Azathioprine with MMF has been associated with improvements in LVEF, reductions in left ventricular end-diastolic volume, and decreases in cardiac biomarkers such as troponin and NT-pro-BNP [52].

### 5.5. Intravenous Immunoglobulin (IVIG)

IVIG is indicated in viral myocarditis, particularly in pediatric patients and in cases of acute myocarditis, due to its immunomodulatory properties. It exerts its effects by regulating T-cell function and reducing pro-inflammatory cytokines such as TNF-α and IL-6. Meta-analyses have demonstrated reductions in mortality and improvements in LVEF with IVIG therapy [9,53].

### 5.6. Interferon-α/Thymomodulin

In a randomized trial involving 38 patients with biopsy-confirmed myocarditis, a 2-year follow-up in patients with dilated cardiomyopathy (DCM) demonstrated improved progression-free survival (PFS): 88% in the interferon group and 66% in the thymomodulin group. These agents may serve as adjuncts to standard therapy in cases of persistent myocardial inflammation [54,55].

### 5.7. Rituximab/Tofacitinib (JAC Inhibitor)

Rituximab has been shown to attenuate the clinical manifestations of myocardial inflammation. There is also a case report indicating successful remission of IVIG-refractory myocarditis following Tofacitinib administration, without adverse effects [52,56].

### 5.8. Experimental Approaches

Emerging therapies include the use of anti-IL-1β monoclonal antibodies, which have demonstrated potential in reducing myocardial inflammation, fibrosis, and adverse remodeling [57].

### 5.9. Biomimetic Nanoparticles

These nanoparticles modulate immune cell activity and are coated with autologous cell membranes (e.g., macrophages or T lymphocytes), enabling targeted delivery to inflamed myocardial tissue. By mimicking native cells, they evade immune detection and can deliver therapeutic agents directly to affected areas, thereby reducing the local immune response [58].

## 6. Conclusions

Myocarditis is a heterogeneous inflammatory disorder with diverse etiologies and pathogenetic mechanisms. Advances in imaging and immunology have changed the traditional diagnostic approach, while a growing understanding of the molecular pathways is reshaping therapeutic strategies. Although supportive management remains the cornerstone of treatment, targeted antiviral, immunosuppressive, and immunomodulatory therapies are expanding the therapeutic landscape. Future research focusing on precision medicine, guided by etiology and immune profiling, is critical for improving patient outcomes and preventing progression to dilated or chronic inflammatory cardiomyopathy.

## Figures and Tables

**Table 1:** Main pathogenetic mechanism involved in acute myocarditis.

Feature	Innate Immunity	Adaptive Immunity
Main Cells	Neutrophils, Macrophages, Dendritic cells, NK	B cells, T cells (CD4^+^, CD8^+^), Plasma cells
Trigger	PAMPs/DAMPs from myocardial injury	Antigen presentation via MHC
Timing	Hours–Days	Days–Weeks
Key Cytokines	IL-1β, IL-6, TNF-α	IL-2, IFN-γ, IL-4, IL-17
Role	Rapid, non-specific defense; debris clearance	Antigen-specific response; memory formation
Dysregulation	Excess inflammation, tissue damage	Autoimmune injury to myocytes

Abbreviations: NK = natural killer, PAMP = pathogen-associated molecular patterns, DAMP = danger-associated molecular patterns, IL = interleukin, TNF = tumor-necrosis-factor.

## Data Availability

No new data were created or analyzed in this study. Data sharing is not applicable to this article.
